# P-1274. Resistance to Metronidazole of Gardnerella in Women with Recurrent Bacterial Vaginosis

**DOI:** 10.1093/ofid/ofaf695.1464

**Published:** 2026-01-11

**Authors:** Ashma Chakrawarti, Erik Swanson, Courtney A Broedlow, Yue Pan, Emily M Cherenack, Nicholas Fonseca Nogueira, Christopher Basting, Lunnarie Acosta, Patricia Raccamariach, Lydia A Fein, Nichole R Klatt, Maria L Alcaide

**Affiliations:** University of Minnesota, Minneapolis, Minnesota; University of Minnesota, Minneapolis, Minnesota; University of Minnesota, Minneapolis, Minnesota; University of Miami Miller School Of Medicine, Miami, Florida; University of Miami, Miami, Florida; Division of Infectious Diseases, Department of Medicine, University of Miami Miller School of Medicine, Parkland, Florida; University of Minnesota, Minneapolis, Minnesota; University of Miami, Miami, Florida; University of Miami, Miami, Florida; University of Miami Miller School of Medicine, Miami, Florida; University of Minnesota Medical School, Minneapolis, Minnesota; Division of Infectious Diseases, Department of Medicine, University of Miami Miller School of Medicine, Parkland, Florida

## Abstract

**Background:**

Bacterial vaginosis (BV) involves a shift from optimal dominance of vaginal *Lactobacillus* species to polymicrobial anaerobic communities, predominantly *Gardnerella*. Despite standard metronidazole treatment, recurrence occurs in 50-80% of cases within one year. While molecular profiling of the vaginal microbiome has advanced, culture-based, *longitudinal* data on *Gardnerella* spp. and its metronidazole susceptibility is limited, particularly across clinically defined recurrent and persistent BV. This study characterized the susceptibility of *Gardnerella* isolates to metronidazole to assess if antibiotic resistance contributes to recurrence.

**Methods:**

Vaginal swabs were collected from women clinically diagnosed with BV by Amsel criteria and treated with 7-day oral metronidazole. BV status was assessed at baseline (T0), one-month (T1), and six-month (T2) after treatment. Recurrent BV was defined as T0 and T2 positive with T1 clearance; persistent BV was positive at all time points. Swabs were cultured anaerobically on NYCIII and V agar media at 37°C for 24-48 hours. *Gardnerella* was presumptively identified by Gram staining, colony morphology, hemolytic patterns on V agar and confirmed by 16S rRNA PCR followed by Sanger sequencing. Metronidazole susceptibility was tested using E-test strips, interpreted using CLSI anaerobic breakpoints, with ATCC 14018 *G. vaginalis* as a control.

**Results:**

Multiple *Gardnerella* species, including *G. leopoldii, G. swidsinskii*, and *G. vaginalis*, were cultured and identified from both recurrent and persistent BV. In recurrent BV, T0 isolates were metronidazole sensitive, while T2 isolates from the same participant showed resistance. Persistent BV isolates demonstrated resistance at both timepoints.

**Conclusion:**

Preliminary findings reveal potential shifts in *Gardnerella* susceptibility after treatment, specifically in recurrent BV with no resistance detectable prior to treatment. These results highlight the importance of culture-based diagnostics and longitudinal monitoring of antimicrobial susceptibility. Continued isolate identification and expanded analysis are underway to better define resistance trends and inform targeted therapeutic approaches for treatment-refractory BV.

**Disclosures:**

Maria L. Alcaide, MD, Gilead: Advisor/Consultant

